# Rapid, simple, and effective strategy to produce monoclonal antibodies targeting protein structures using hybridoma technology

**DOI:** 10.1186/s13036-023-00345-9

**Published:** 2023-03-30

**Authors:** Atsumi Sakaguchi, Yoichiro Tanaka, Eiki Shoji, Teppei Takeshima, Rina Sakamaki, Takao Matsuba, Yasuyuki Kurihara

**Affiliations:** 1 Laboratory of Molecular Biology, Faculty of Engineering, Yokohama National University, 79-5, Tokiwadai, Hodogaya, Yokohama, Kanagawa 240-8501 Japan; 2 Biomaterials Analysis Division, Open Facility Center, Tokyo Institute of Technology, Midori, Yokohama, Kanagawa Japan; 3 Instrumental Analysis Center, Yokohama National University, Hodogaya, Yokohama, Kanagawa Japan; 4 Department of Urology and Renal Transplantation, Yokohama City University Medical Center, Yokohama, Japan; 5 Bioscience Division, Tosoh Corporation, Ebina, Kanagawa Japan

**Keywords:** Monoclonal antibody, Flow cytometer, Conformation-specific antibody, ELISA, Hybridoma technology

## Abstract

**Background:**

Monoclonal antibodies are essential in life science research and developing antibody drugs and test drugs. Various methods have been developed to obtain monoclonal antibodies, among which hybridoma technology continues to be widely used. However, developing a rapid and efficient method for obtaining conformation-specific antibodies using hybridoma technology remains challenging. We previously developed the membrane-type immunoglobulin-directed hybridoma screening (MIHS) method, which is a flow cytometry-based screening technique based on the interaction between the B-cell receptor expressed on the hybridoma cell surface and the antigen protein, to obtain conformation-specific antibodies.

**Results:**

In this study, we proposed a streptavidin-anchored ELISA screening technology (SAST) as a secondary screening method that retains the advantages of the MIHS method. Anti-enhanced green fluorescent protein monoclonal antibodies were generated as a model experiment, and their structural recognition abilities were examined. Examination of the reaction profiles showed that all monoclonal antibodies obtained in this study recognize the conformational epitopes of the protein antigen. Furthermore, these monoclonal antibodies were classified into two groups: those with binding activities against partially denatured proteins and those with complete loss of binding activities. Next, when screening monoclonal antibodies by the MIHS method as the first screening, we found that monoclonal antibodies with stronger binding constants may be selected by double-staining for hybridomas with fluorescently labeled target antigens and fluorescently labeled B cell receptor antibodies.

**Conclusions:**

The proposed two-step screening method, which incorporates MIHS and SAST, constitutes a rapid, simple, and effective strategy to obtain conformation-specific monoclonal antibodies generated through hybridoma technology. The novel monoclonal antibody screening strategy reported herein could accelerate the development of antibody drugs and antibody tests.

**Supplementary Information:**

The online version contains supplementary material available at 10.1186/s13036-023-00345-9.

## Highlights

・MIHS-SAST screening generates mAbs that target protein structures.

・One-fourth of the mAbs recognized stereospecific epitopes of the protein.

・Two fluorometric parameters aided in selecting antibodies with strong binding constants.

**Supplementary Information:**

The online version contains supplementary material available at 10.1186/s13036-023-00345-9.

## Introduction

Monoclonal antibodies (mAbs) have high affinity and binding specificity for target molecules and are widely used in life science applications, such as in immunoassays and flow cytometry [1]. Additionally, mAbs enable use as biosensor for diagnosis and detection of various emerging infectious diseases, foods, and illicit drugs [2]. Since 1975, when Koehler and Milstein [3] reported that mAbs could be produced via hybridoma technology, which fuses B cells with myeloma cells, many laboratories and companies have used hybridoma technology to produce useful mAbs. Subsequently, various alternative and innovative methods were developed for mAb production. The in vitro phage display method enabled the rapid production of mAbs without using immunized animals. However, this method needs further improvement, including better antibody binding constants and procedures for building and maintaining large phage libraries of optimal diversity [4–6]. Several methods have been developed to obtain mAbs by immortalizing B cells selected from the blood cells of patients recovering from an infection or by cloning immunoglobulin genes from similarly selected cells and genetically engineering them to produce antibodies [7–9]. This technology has substantially contributed to the development of neutralizing antibodies against infectious diseases, such as Covid-19 [10]. However, only a few laboratories currently have access to these technologies [7].

Hybridoma technology is methodologically simple and can be implemented in any laboratory. Moreover, this technology not only takes advantage of the in vivo mechanisms (e.g., genetic recombination and somatic hypermutation) that enable the generation of diverse antibodies [5, 11] but also enables the generation of mAbs with strong and specific binding abilities.

Hybridoma technology has high potential and versatility; however, the repeated hybridoma screening and cloning process and cultivation of multiple positive clones are laborious, costly, and time-consuming. Furthermore, despite considerable efforts, this approach does not always produce high-quality mAbs with desired applications. Another major challenge in mAb production is that most of the obtained antibodies only recognize the linear epitopes of the antigen, and antibodies that recognize the physiological structure of the antigen cannot be efficiently obtained [5]. Antibodies for therapeutic drug testing must essentially recognize the physiological structure of the antigen to target biological substances. Therefore, to obtain mAbs for drug testing and support the development of therapeutic drugs for emerging infectious diseases, a rapid, simple, and effective strategy to produce mAbs that recognize the conformational epitopes of a target protein is required.

B-cell receptor (BCR), a type of transmembrane immunoglobulin, has recently been gaining increasing attention owing to its applicability as a tag for screening structure-recognizing antibodies [12–17]. Hybridomas not only secrete soluble antibodies but also express BCRs on their cell membranes. Because the BCR and its corresponding secreted antibody share the same antigen-binding specificities, hybridomas producing antibodies that bind to target antigens can be obtained by screening them using the binding BCRs as indicators. The membrane-type immunoglobulin-directed hybridoma screening (MIHS) method involves three steps: (1) introduction of a fluorescent-labeled antigen into the culture medium, (2) binding of the antigen to the BCR on the hybridoma cell surface, and (3) selection of fluorescent-labeled hybridomas via flow cytometry (FCM) [14, 15]. FCM enables the simultaneous screening of numerous cells and single-cell sorting in a short time, thereby allowing tedious and time-consuming screening and cloning to be completed in a single operation. Another important advantage of the proposed method is that it can selectively generate conformational epitope-specific mAbs, as screening is performed by binding antigens with three-dimensional structures to BCRs in a hybridoma medium.

As some hybridomas selected by MIHS were pseudo positive clones, secondary screening must be performed [14, 15]. The secondary screening method should retain the advantages of the MIHS method, which recognizes the conformational epitope of the antigen. Furthermore, as MIHS selects only a few hundred clones of positive cells from hundreds of thousands of hybridomas, a simple and relatively high-throughput secondary screening method must be used for selection.

Immunostaining is a method to detect the localization of intracellular or cell membrane proteins and can be used for hybridoma screening. Fixation during immunostaining or the formation of protein complexes with the target antigen protein may denature the antigen structure or hide the epitope. Furthermore, immunostaining is labor-intensive and not suitable for high-throughput screening. In the sandwich ELISA method, because the antigen is not directly attached to the plate, antibodies that react with the antigen and retain their original structure are obtained. Moreover, the procedure is simple and can be performed without using special equipment. However, a major disadvantage of this method is that it requires paired and secondary antibodies [18]. Immunoprecipitation is an excellent method for obtaining antibodies that recognize conformational epitopes of antigens; however, it is costly to use this method for high-throughput screening. To resolve these problems, we devised streptavidin-anchored ELISA screening technology (SAST) as a secondary screening method for hybridomas and confirmed its usefulness; however, the concept of SAST is not novel [19–21]. In this study, we identified the novel and useful properties enabling this method to be used to screen hybridomas producing conformational epitope-specific mAbs. Owing to this new utility, we have named this method SAST.

The SAST method involves anchoring a biotin-labeled antigen protein to an ELISA plate with immobilized streptavidin, following which the ability of the antibody to bind to the antigen is screened. The binding of biotin to streptavidin has the highest binding constant in nature and has been used in a wide range of life science studies. There are two strategies (chemical and enzymatic) for introducing biotin into antigen proteins. In chemical methods, biotin is introduced into the amino, thiol, and aldehyde groups of amino acids, such as lysine and cysteine [16, 17, 20]. However, this approach is not suitable for antigen production because biotin can modify antigenicity. However, in enzymatic method, a biotin-protein ligase (BirA) introduces biotin into the lysine residue of a specific 15 amino acid residue biotin acceptor peptide (BAP) sequence. In this method, biotin can be introduced into a BAP-fused recombinant protein without epitope modification. Furthermore, biotin labeling with BirA is efficient and inexpensive when BirA is produced via genetic engineering [21], thereby making it a useful biotin-labeling method. When the biotinylated antigen protein is fixed to a streptavidin-coated ELISA plate, the antigen is fixed away from the plate, and the three-dimensional structure of the antigen protein is expected to be retained. Therefore, screening hybridomas that bind to the conformational epitopes of antigen proteins could enable the development of conformational epitope-specific mAbs, which is a characteristic of the MIHS method. Moreover, as the protocol of the SAST method is nearly the same as that of the ordinary ELISA method, antibodies can be easily screened.

Herein, we report a two-step hybridoma screening method with MIHS as the primary screening method and SAST as the secondary screening method that can rapidly and selectively obtain conformation-specific antibodies with high binding affinity. This two-step screening method is expected to be useful in developing antibody drugs and diagnostic agents for emerging infectious diseases.

## Materials and methods

### Chemicals

All chemicals were purchased from Fujifilm Wako Pure Chemicals (Osaka, Japan). Restriction enzymes used in this study were obtained from Takara Bio Inc (Shiga, Japan).

### Enhanced green fluorescent protein (EGFP) production

The experiments were conducted using His-EGFP-pColdII, an expression vector that produces recombinant EGFP, as reported in a previous study [15]. The synthesized DNA sequences for BAP [21] and 6xHis (Eurofins) were inserted into the Pst I-Xba I site of pCold III. Furthermore, a sequence encoding EGFP was inserted into the BamHI-PstI site of this vector to prepare EGFP-BAPHis-pCold III. DNA sequencing confirmed this DNA sequence. The recombinant proteins were purified as previously described [15].

### *In vitro* biotinylation of recombinant EGFP

The production of recombinant BirA enzyme and biotin labeling of the purified EGFP-BAPHis protein were conducted in a manner similar to that described by Fairhead and Howarth [21]. For biotin labeling, 1 µmol Bir A and 150 µmol D-biotin were added to 500 µL PBS containing 5 mM MgCl_2_, 2 mM ATP, and 20 µmol EGFP-BAPHis protein. The mixture was then gently stirred at 30 ℃. After 1 h, 150 µmol D-biotin and 1 µmol BirA were added, and the mixture was gently stirred for an additional hour. The reaction mixture was then dialyzed twice with 1 L of PBS to remove unreacted D-biotin.

A supershift assay was then performed to confirm the D-biotin labeling of the EGFP-BAPHis protein [21]. Briefly, 2.5 µL of 5 × sample buffer (312.5 mM Tris–HCl pH6.8, 10% sodium dodecyl sulfate, 50% glycerol, 25% 2-mercaptoethanol, 0.1% Bromophenol Blue) was added to 12.5 µL of 10 µM biotin-labeled EGFP-BAPHis protein solution and boiled for 5 min. After cooling, 1 µL of streptavidin solution adjusted to 2 mg/mL with PBS was added and incubated for 5 min. The sample was then separated using 12% SDS-PAGE, and the gel was subjected to Coomassie Briliant Blue staining (250 mg Coomassie Brilliant Blues R250, 10 mL acetic acid, 45 mL methanol, 45 mL distilled water) to confirm the supershift owing to the binding of streptavidin to the biotin-labeled antigen.

### Hybridoma and MIHS screening

The hybridoma used in this study was prepared by immunizing mice with anti-His-EGFP, followed by cell fusion, aliquoting the hybridoma cells into tubes, and cryopreservation. Both our previous study [15] and the present study used cells that were stocked at the same time. After culturing, hybridoma cells were screened using the MIHS method. Briefly, 2 nmol of His-EGFP was added to 1 mL of 5 × 10^5^ hybridoma cell culture medium, following which the cells were cultured at 37 ℃ and 5% CO_2_ for 2 h. The cells were then washed three times with serum-free RPMI 1640, and EGFP-positive cells were seeded individually using a MoFlo Astrios cell sorter (Beckman Coulter Inc, CA), as described in a previous study [15].

### SAST and denatured SAST

Streptavidin (200 ng/50 µL in PBS) was coated into the wells of a 96-well ELISA plate (Sumitomo Bakelite, Tokyo, Japan), shaken gently with a microplate mixer, and left at 4 ℃ overnight. The next day, the same volume of 10% skim milk in TBST was added to the wells (5% final skim milk concentration), and the plate was shaken for 1 h at room temperature. After discarding the solutions from the wells, biotin-labeled recombinant EGFP (200 ng/50 µL) was added to each well and shaken for 10 min. The wells were washed five times with PBS, the hybridoma culture supernatant was diluted to 1:4 with PBS, and the mixture was shaken for 1 h. After five washes with PBS, 50 µL of goat anti-mouse IgG (H + L)-HRP (Medical and Biological Laboratories, Tokyo, Japan) diluted 10,000-fold with TBST was added, and the plate was shaken for 1 h. The plate was washed thrice with PBS, and bound antibodies were detected using the TMB Microwell Peroxidase Substrate System (Seracare, MA). Absorbance at 405 nm was measured using a PowerScan HT multiwell plate reader (Sumitomo Pharma Promo, Osaka, Japan).

For dSAST, heat-denatured biotin-labeled recombinant protein antigens were used. Biotin-labeled EGFP-BAPHis protein (20 µg/10 mL in TBST) was boiled for 3 min, and the disappearance of green fluorescence was confirmed. The denatured protein antigen was immobilized on a streptavidin-coated ELISA plate, following which the same procedure was performed for SAST.

### Western blotting (WB)

WB was used to confirm the binding specificities of antibodies to the antigen and identify isotypes, as described previously [15].

### Determination of the binding constants

Using the Biacore 8 K instrument (Cytiva, Tokyo, Japan), the association and dissociation rate constants (*k*_*a*_ and *k*_*d*_, respectively) and dissociation constants (*K*_D_ = k_d_/k_a_) of the anti-EGFP mAbs were calculated. Rabbit anti-mouse IgG polyclonal antibodies were immobilized on a Series S Sensor Chip CM5 (Cytiva, Tokyo, Japan). The anti-EGFP mAb was captured on the sensor chip. Recombinant EGFP was diluted with running buffer in two dilution series (5 µM to 312.5 nM and 500 nM to 31.25 nM in a two-fold dilution) and analyzed in duplicate using the multi-cycle kinetics in the appropriate dilution series. After each cycle, the sensor surface was regenerated using a glycine–HCl solution at pH 1.7. All analyte samples were diluted in 1 × HBS-EP + running buffer.

### Reversed immunoprecipitation

A mixture of 10 µL streptavidin magnetic beads (JSR Life Sciences, CA) and 5.2 µg biotin-labeled EGFP-BAPHis recombinant protein in 500 µL PBS was periodically inverted at room temperature for 20 min. The beads were attracted to the magnet to remove the supernatant and washed twice with 500 µL PBS. Subsequently, 100 µL of the hybridoma culture supernatant and 50 µL of PBS were added, and the mixture was gently spun at 4 ℃ for 2 h. The beads were adsorbed on a magnet, and the supernatant was removed, following which the beads were washed twice with 500 µL of PBS. The samples were then suspended in 20 µL of 1 × SDS sample buffer and boiled for 3 min, and the supernatants were separated by SDS-PAGE. The immunoprecipitation of the antibody was confirmed using WB with goat anti-mouse IgG (H + L)-HRP antibodies (Medical and Biological Laboratories, Tokyo, Japan) or goat anti-mouse IgM µ chain-HRP antibodies (MilliporeSigma, MO).

### Statistical analysis

Unpaired Student’s t-tests and other statistical evaluations were analyzed using descriptive statistics in the Excel software in Microsoft Office 365 (Microsoft, WA). Statistical significance was set at *p* < 0.05.

## Results

### Principle and reproducibility of SAST

**Fig. 1:**
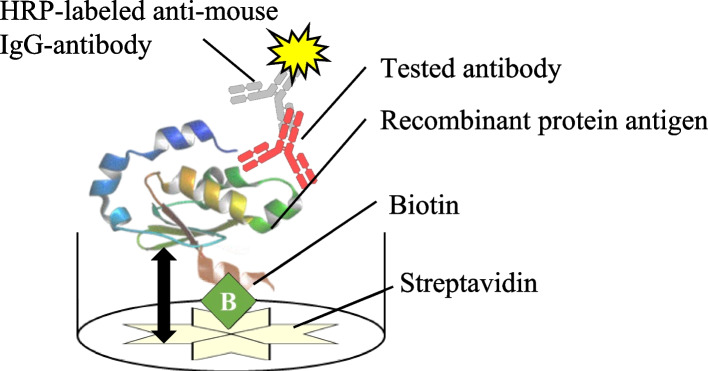
Concept of the SAST method. After applying streptavidin to the ELISA plate, biotinylated antigen protein was fixed. The distance between the bottom of the plate and the antigen protein was expected to be at least 10 nm (both arrows), which could maintain the undenatured structure of the protein. Subsequently, the tested antibodies were applied, the unbound antibodies were washed away with PBS, and HRP-conjugated anti-mouse IgG secondary antibodies were added to the wells. After washing the wells, the bound secondary antibodies were visualized as described in the Materials and Methods. This method is expected to allow the mAb screening of antigens with their undenatured structures

The basic strategy of SAST is shown in Fig. 1. A BAP was fused to the antigen protein, and biotin was introduced into the lysine residue in the tag sequence by BirA. Streptavidin is efficiently absorbed by ELISA plates and does not lose its biotin-binding ability. Therefore, biotinylated proteins can be efficiently immobilized on streptavidin-coated ELISA plates [19, 20]. The apparent size of the streptavidin tetramer is 5 nm [22], and assuming a contour size of 0.4 nm per amino acid [23], the antigen protein was fixed at an estimated distance of at least 10 nm from the ELISA plate. This presumably allows the antigen protein to be fixed without interference from streptavidin or the bottom of the ELISA plate, while maintaining its conformational epitopes [19]. Furthermore, the tested antibodies were added, and antigen–antibody binding was detected as described in the Materials and Methods section. The basic method of operation was the same as the usual direct and indirect ELISA methods; therefore, it can be easily performed in any laboratory.

First, the efficiency of biotin incorporation into the antigen protein was evaluated via a supershift assay. As shown in Fig. S1, the addition of streptavidin to the EGFP protein did not supershift the protein band (lane 4). However, in the case of biotinylated EGFP protein, the bands of the labeled protein disappeared and supershifted (lane 5), indicating that the protein can be efficiently biotinylated by BirA. We then examined the reproducibility of the SAST method using the culture supernatants of 94 anti-EGFP mAb clones, as described in a previous study [15]. The comparison of values observed by the two independent experiments (Fig. S2) confirmed the low degree of dispersion and high reproducibility of SAST (linear approximation equation y = 0.9682x).

### Hybridoma screening of anti-EGFP mAbs using MIHS and SAST

To demonstrate the applicability of SAST, we generated anti-EGFP mAbs as a model experiment. The target antigen was selected for two reasons. First, the physiological structure of EGFP was confirmed using green fluorescence. Second, we used cryopreserved aliquots of hybridomas produced using immunization and cell fusion, as described previously [15]. This enabled us to compare the efficiencies of antibody acquisition in the present and previous experiments.

One of the cryopreserved fused cells was thawed and cultured in the HT medium for 3 days. The cells were labeled with recombinant EGFP and analyzed via flow cytometry. The mode of hybridoma labeling intensity (H-LI) of the unlabeled hybridoma was regarded as background fluorescence, and labeled cells showing a fluorescence intensity of 3 × [H-LI] or higher were considered positive cells. As shown in Fig. S3, approximately 1% of the cells was labeled and sorted into 96-well plates, with one positive cell per well. The culture supernatant of 273 wells grown as a colony was subjected to secondary screening via the SAST method, and 63% (172/273 wells) was positive (Table 1). The previously reported positive acquisition efficiencies of ELISA-WB screening (EW) and MIHS-WB screening (MW) were 1.8% and 15.2%, respectively [15]; however, the MIHS-SAST screening (MS) method acquired positive cells from secondary screening at a higher rate.

**Table 1:** Comparison of the efficiency of mAb acquisition by EW, MW and MS combinations

First Screening	Secondary screening	Abbreviation	Positive Clone % (positive/analyzed)	
ELISA	Western blotting	EW	1.8 (24/1300)	(14)
MIHS		MW	15.2 (57/375)	
	SAST	MS	63.0 (172/273)	This work

### Antigen-binding specificity and isotype determination by WB

**Fig. 2:**
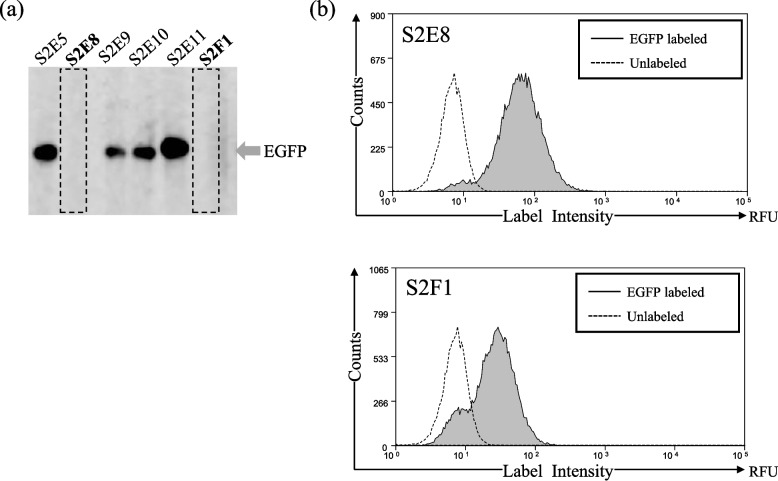
Western blotting reactivities and fluorescent labeling of hybridomas of the MS clones. **a** mAbs obtained in combination with MS screening were subjected to WB against *Escherichia coli*-expressed EGFP. A subset of them is shown in the figure, and the arrows indicated the EGFP signal. Some clones showed a strong single signal, while S2E8 and S2F1 did not give a signal. **b** Two hybridomas (S2E8 and S2F1) secreting mAb that did not give a signal in WB were labeled with EGFP and analyzed via FCM. Areas marked in gray indicate hybridomas labeled with EGFP. RFM, relative fluorescence units

Of the 172 clones that tested positive in SAST, 72 antibodies were analyzed using WB for reactivity against the EGFP-BAPHis protein produced by *Escherichia coli*. Consequently, seven clones (9.7%) showed non-specific reactivity, and 32 clones (44.4%) showed positive bands with high specificity. Notably, 33 clones did not show positive reactions (representative WB images are shown in Fig. 2a). However, as strong H-LI was observed even in WB-negative clones, as observed in S2E8 and S2F1 (Fig. 2b), we found that some clones did not react to WB, although the antigen did bind to the BCR on the hybridoma. Because these mAbs seemed to be conformational epitope -specific mAbs, we examined the H-LIs of 65 hybridoma clones producing 32 antibodies that specifically reacted with WB and 33 antibodies that did not positively react with WB for binding to the EGFP antigen by FCM. The H-LI of the hybridoma without EGFP (negative control) was 5.56, whereas the H-LI measurement of 65 clones showed a wide range from 17.98 to 196.84. These results corroborated the finding that positive clones with more than three times the H-LI of the negative control were selected via MIHS screening. Sufficient H-LI was also observed in the 33 clones that did not show a positive reaction in WB, indicating that these clones also produced antibodies that reacted with EGFP. These results are shown in the additional file.

**Fig. 3:**
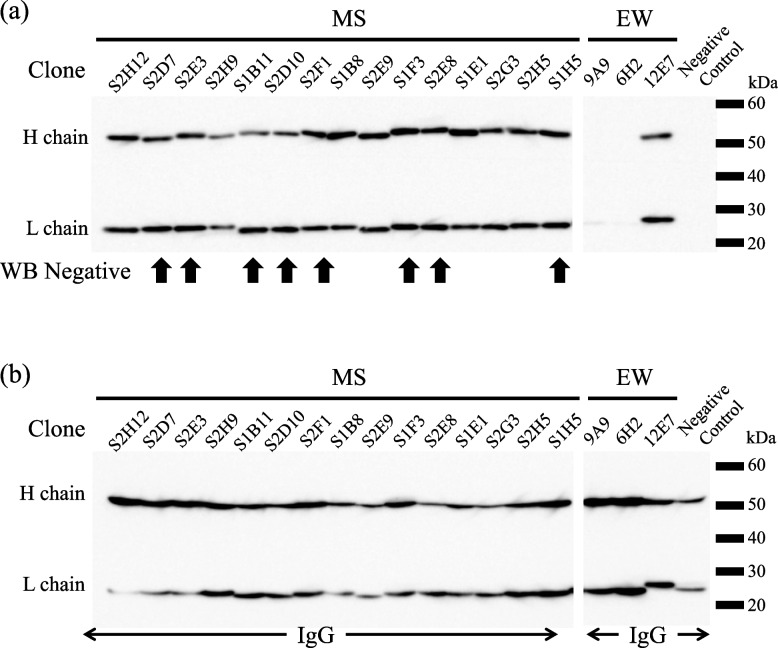
Immunoprecipitation and Ig class determination of MS clones. **a** Immunoprecipitation was performed using 15 representative clones obtained by MS combination, and the precipitants were subjected to SDS-PAGE followed by WB with HRP-anti-mouse IgG antibody. The samples indicated by arrows are antibodies that did not give signals in WB; however, all mAbs, including these, were antibodies that could be used for immunoprecipitation. For comparison, the results of immunoprecipitation using antibodies obtained by EW are shown; however, only one of the three mAbs could be used for immunoprecipitation. **b** Ig class determination. After electrophoresis of the culture supernatant of the hybridomas of the MS clone by SDS-PAGE, WB was performed with HRP anti-mouse Ig antibody. The results showed that all mAbs used in the immunoprecipitation test were from the IgG class, as they all showed bands of H chain of approximately 55 kD and L chain of approximately 25 kD. The Ig class results for the other MS clones are shown in the additional file. The mAbs of the clones obtained by EW were also from the IgG class

To demonstrate that the mAbs obtained by MS were antibodies that recognize the structure, immunoprecipitation was performed using eight mAbs for which no bands were detected by WB and seven mAbs for which specific bands were detected (Fig. 3a). For comparison, three anti-EGFP mAbs obtained using the EW combination were used for immunoprecipitation. When conducting immunoprecipitation, antibodies are usually immobilized on beads, and their ability to bind to antigens is examined. However, the ability to bind proteins A and G depends on the antibody class. In this study, biotinylated EGFP antigens were adsorbed onto streptavidin beads to eliminate the variations in their binding ability, and the antibodies bound to the protein were confirmed via WB to determine their immunoprecipitation ability. Two of the three clones obtained using the conventional EW combination could not be immunoprecipitated. In contrast, all clones obtained via the MS combination were immunoprecipitated. Among these, eight mAbs that did not react in the WB assay are indicated by the arrows in Fig. 3a. These results strongly suggest that the mAbs obtained by MS recognized the conformational epitopes of the antigen, as the mAbs could be immunoprecipitated regardless of WB reactivity.

Subsequently, the immunoglobulin class of the mAbs was determined by WB as previously described [15]. Sixty-five clones of the hybridoma culture supernatants obtained by MS, excluding clones showing multiple bands in WB analysis, were analyzed using anti-mouse IgG-HRP and anti-mouse IgM-HRP antibodies. Dataset are shown in the additional file, and representative data are shown in Fig. 3b. All mAbs obtained by MS combination belonged to the IgG class, comprising H chains of ~ 55 kDa and L chains of 25 kDa.

### Characterization of the conformational recognition ability of mAbs

**Fig. 4:**
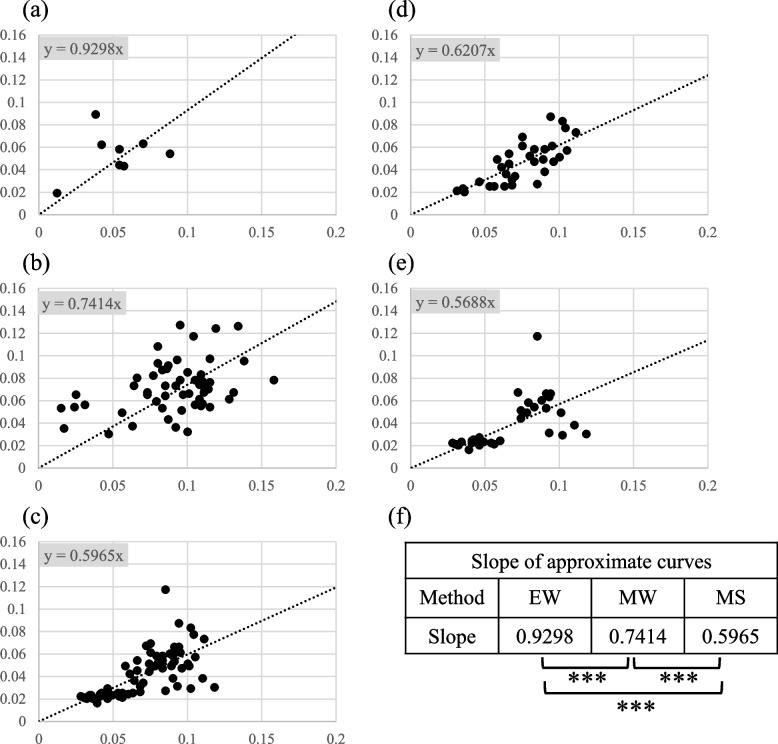
Correlation between ELISA and SAST experimental values. **a** ELISA values of eight clones obtained by the EW method were plotted on the vertical axis, and SAST values were plotted on the horizontal axis, resulting in an approximate equation with small dispersion and a slope of approximately 1. **b** For the 54 clones obtained by the MW method, the variation was small, and an approximate equation with a slope of 0.7414 was obtained, indicating that the SAST experimental value was slightly higher than the ELISA experimental value. **c** ELISA and SAST values of 65 clones obtained by MS combination were plotted as in (**a**), showing that the dispersion was small. The slope was 0.5965, indicating that the SAST experimental values were higher than the ELISA experimental values. **d** ELISA and SAST values of 32 WB positive clones of MS were plotted, and an approximate equation was obtained. (e) ELISA and SAST values of 33 WB negative clones of MS were plotted to obtain an approximate equation. Note that the slope of the approximate equation of (d) was higher than that of (e); however, there was no significant difference between the two values. (f) Student's t-test analysis confirmed no significant difference between ELISA and SAST absorbance in EW; however, SAST experimental values were significantly higher than those of ELISA in MW and MS. ***; *p* < 0.01

As the results of SAST, WB, and immunoprecipitation suggested that the mAbs obtained using MS recognized conformational epitopes of the antigen protein, we compared the experimental values of ELISA and SAST for further characterization of the conformation specificities of mAbs. Although non-covalent binding is generally involved in the binding of proteins to ELISA plates, it is known that hydrophobic interactions play a major role, particularly in aqueous solvents. Therefore, when a protein is immobilized on an ELISA plate, the hydrophobic region of the protein adheres to the bottom of the plate, which is considered to modify protein structure. However, as antigens used in SAST are expected to have a physiological structure, we used mAbs obtained by MS, MW, and EW to examine the difference in reactivity when using different screening tags in ELISA and SAST. First, ELISA was performed with 65 mAbs derived from MS. Subsequently, ELISA and SAST were performed for eight mAbs derived from EW clones and 54 mAbs from MW clones. All the observed values are shown in the Additional file 1; Table S1. The incremental absorbance was obtained by subtracting the background values without primary antibody from the measured values (ELISA; 0.0538, SAST and dSAST; 0.0617) and plotted for each clone (Fig. 4). Next, in order to compare the binding of mAb to the antigen by the three methods, an approximate straight line was obtained for these plots. assuming an approximate straight line of y = *a*x + *b,* the values of* b* for the three methods were very different, affecting the slope (*a*). The *R*^2^ value was also small, and these approximate straight lines were considered to be inappropriate in this analysis. The incremental absorbances can be obtained by subtracting the background values. Since the incremental absorbance should be zero if the antigen and antibody do not react, it should be reasonable to assume an approximate straight line with y = *a*x. The calculated approximate line between the ELISA and SAST values for the EW mAbs was y = 0.9298x (Fig. 4a). This suggested that each mAb obtained using EW bound to the same or similar epitope structures between the two methods (Fig. 4f). Subsequently, mAbs produced by MW (54 clones) and MS (65 clones) were analyzed similarly, and the results indicated a linear approximation of y = 0.7414 × and y = 0.5965x, respectively (Fig. 4b, c). These showed that, unlike those of EW, the clones obtained by MW and MS responded more strongly to the SAST antigen structure than ELISA antigen. Student's t test for significance of the slope values (*a*) obtained by the three methods showed that all combinations were significantly different (*p* < 0.01) (Fig. 4f). This means that mAbs obtained by MW and MS recognize different antigen epitopes in ELISA and SAST, and that MS can obtain mAbs that bind more strongly to the antigens with undenatured antigens in SAST. In addition, some of the MS clones had mAbs that reacted with WB and others did not. These were plotted and analyzed in the same way (Fig. 4 d, e), and the mAbs that did not react with WB had a smaller slope (*a*) of the approximate line, although not significantly different, suggesting that they might also be recognizing more nondenaturing antigen structures.

**Fig. 5:**
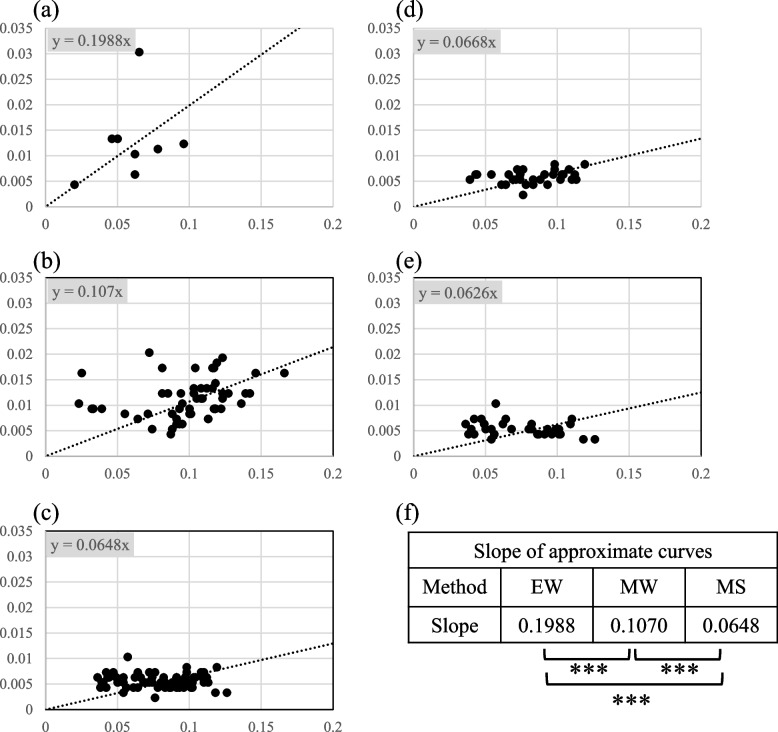
Correlation between SAST and dSAST experimental values. **a** dSAST values of the eight clones obtained by the EW method were plotted on the vertical axis and the SAST values on the horizontal axis; an approximate equation between the two data sets was calculated, yielding a slope of 0.1988. This indicated that the dSAST values were lower than the SAST values; however, these mAbs had reactivity to the antigen after thermal denaturation. **b** dSAST and SAST values were plotted for the 54 clones obtained by the MW method as in (**a**). The approximate equation showed a slope of 0.107, which was smaller than the slope of EW, indicating that the reactivity between mAb and antigen was reduced owing to thermal denaturation. **c** dSAST and SAST values of 65 clones were obtained by MS combination as in (**a**), and the slope of the approximate equation was 0.0648. This indicates that the antigenicity recognized by MS antibodies was mostly lost after thermal denaturation. **d** ELISA and SAST values of 32 WB positive clones of MS were plotted, and an approximate equation was obtained. **e** ELISA and SAST values of 33 WB negative clones of MS were plotted, and an approximate equation was obtained. Note that the slope of the approximate equation of (**d**) was slightly higher than that of (**e**); however, although there was no significant difference between the two values. **f** Using Student's t-test, we examined the significance between the slopes of the three plots; no statistically significant difference was observed between EW and MW; however, the slope was slightly lower for MS. A significant difference was observed between EW and MS, indicating that mAbs recognizing epitopes sensitive to thermal denaturation were obtained in MS compared to EW. ***: *p* < 0.01

SAST and dSAST values were then compared for mAbs obtained from EW, MW, and MS. As shown in Fig. 4, the incremental absorbances were plotted for each method (Fig. 5). Although the incremental absorbances were greatly reduced due to the thermal denaturation of the antigen, the mAbs obtained by EW had the highest dSAST values and showed little reaction with MS mAbs. Furthermore, there was a significant difference in the slope of the approximate straight line for the three methods (*p* < 0.01; Fig. 5f), indicating that MS and MW recognize epitopes more strongly affected by thermal denaturation. Furthermore, as shown in Fig. 4, when mAbs that reacted with WB were compared with mAbs that did not react with WB in MS clones, the slope (*a*) was smaller for mAbs that did not react with WB. However, the difference was insignificant, suggesting that mAbs that did not react with WB were more strongly affected by thermal denaturation.

All mAbs obtained by MS were divided into four groups based on their reactivity, as summarized in Table 2. All mAbs obtained by MS recognized the functional structure of antigens because they showed a positive result upon immunoprecipitation and reacted with MIHS and SAST. However, some of these mAbs reacted with one or both WB and ELISA (Groups B, C, and D), suggesting that the different sites of antigen protein denaturation in WB and ELISA led to diversity in mAb reactivity. Notably, conformational epitope-specific and highly denaturation-sensitive mAbs were obtained at a frequency of approximately 25% (Group A).

**Table 2:** Groups of ELISA, SAST, and dSAST reactivity patterns of mAbs obtained from MS combination

Group	Native Structure		Denatured Structure			Number of Clones
	MIHS	SAST	ELISA	WB	dSAST	
A	〇	〇	×	×	×	15
B	〇	〇	〇	×	×	18
C	〇	〇	×	〇	×	6
D	〇	〇	〇	〇	×	26

### Estimation of parameters predicting binding strength

**Fig. 6:**
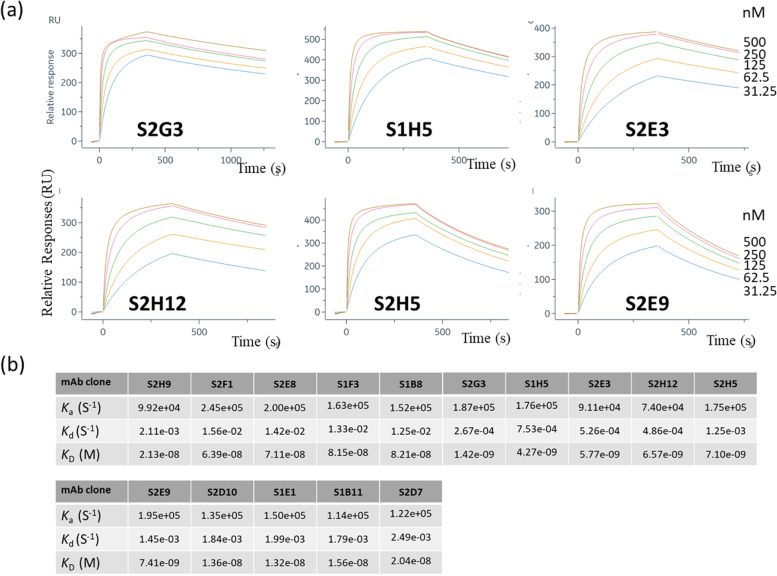
Binding affinities of MS mAbs shown by surface plasmon resonance. **a** Sensograms showing the concentration-dependent bindings of six representative mAbs to the antigen protein (**b**) Surface plasmon resonance yielded measures of the association constant (*k*_a_), dissociation constant (*k*_d_), and affinity (*K*_D_)

The binding strength of an antibody to a target molecule is an important parameter for assessing antibody quality. Surface plasmon resonance experiments were performed using a BIAcore 8 K, and the binding constants (*K*_*D*_) of 15 mAbs obtained by MS were determined. The results showed that the antibodies had a wide range of binding constants (14.3–822 nM) between clones (Fig. 6b and Table 3A and the sensorgrams were shown in Fig. 6a and Fig. 4S). When screening mAbs, selectively obtaining mAbs with a high binding strength is also important. It would be more useful to have clues to selectively obtain high-binding mAbs when screening hybridomas.

**Table 3:** Evaluation of parameters of various structural recognition properties of selected mAbs obtained by MS combination

Clone Name	*K*_D_ (nM)	Hybridoma		BCR	H-LI per unit BCR	
		H-LI (RFU)	Hybridoma Group	B-LI (RFU)	(H-LI)/(B-LI)	Group
S2G3	14.2	60.86	lH	196.84	0.31	hU
S1H5	42.7	33.84	lH	225.39	0.15	lU
S2E3	57.7	114.50	hH	354.01	0.32	hU
S2H12	65.7	188.15	hH	424.08	0.44	hU
S2H5	71.0	44.37	lH	143.50	0.31	hU
S2E9	74.1	114.50	hH	405.36	0.28	hU
S1E1	132	46.42	lH	1046.18	0.04	lU
S2D10	136	69.68	lH	246.69	0.28	hU
S1B11	156	76.27	lH	188.15	0.41	hU
S2D7	204	179.85	hH	1046.18	0.17	lU
S2H9	213	95.59	lH	636.68	0.15	lU
S2F1	639	171.91	hH	1570.65	0.11	lU
S2E8	711	50.80	lH	729.03	0.07	lU
S1F3	815	100.00	hH	1145.05	0.09	lU
S1B8	821	157.06	hH	1253.25	0.13	lU

While the *K*_*D*_ of the mAb was the binding property of the antibody itself, the parameter used for screening (H-LI) was the fluorescence intensity obtained by fluorescent-labeling the binding of the antigen to the BCR on the hybridoma cell membrane. The H-LI value was considered to depend on the binding properties of the BCR and the number of BCR. Therefore, to confirm the number of BCRs, the BCRs of the 15 hybridoma clones were labeled with fluorescent anti-mouse Ig antibodies, and the fluorescence intensity of the BCRs (B-LI) was measured using FCM. Subsequently, (H-LI)/(B-LI) was calculated to obtain the H-LI per unit BCR (U-LI). Based on this value, we considered that the binding intensity of BCRs to the antigen could be estimated independently of the number of BCRs. The results are presented in Table 3, with the U-LI values ranging from 0.04–0.44. For clarity, the average of all U-LI values was determined (average = 0.22), and each clone was classified into hU with a higher U-LI than the average and lU with lower values. As shown in Table 3, clones with strong *K*_*D*_ tended to have a high U-LI, and clones with weak *K*_*D*_ tended to have a low U-LI From these results, it was inferred that clones with high binding strengths could be obtained by selectively obtaining clones with low B-LI and high H-LI using both antigen and BCR labels in hybridoma screening.

## Discussion

Antibodies that recognize the functional structure of proteins are very useful for research and therapeutic purposes in life sciences. In this paper, we report the development of a simple two-step screening system that enables the acquisition of mAbs that recognize conformation specific epitopes of the antigens. mAbs can generally be classified into two categories based on their recognition epitope structures [24]. One is a linear epitope antibody that recognizes the alignment of amino acids, and the other is a conformational epitope (stereo-specific) antibody that recognizes the conformational structure of proteins. However, in practice, it is difficult to classify antibodies into these two categories based on their reaction characteristics. For example, mAbs that can be used for immunoprecipitation are generally considered to be antibodies that recognize conformational epitopes; however, if the epitope is a linear or random region of the protein structure, linear epitope-specific mAbs that recognize the alignment of amino acids can also be used for immunoprecipitation. When peptide antigens are used as immunogens, antigen sequences are typically designed for the terminal regions of proteins, so that they can be used in a wide range of applications, such as immunoprecipitation [25]. This is because the terminal regions of the protein are exposed to the solvent and are more likely to adopt a random structure, increasing the probability of obtaining antibodies that can be used for immunoprecipitation. Therefore, it is easy to imagine the two classifications; however, experimentally distinguishing whether the epitope of the mAb is a linear epitope or a conformational epitope is difficult.

We propose three classifications based on the reaction properties of antibodies that can help infer the applications of mAbs.

### Class 1: mAbs for the epitopes of intact structures

This class of mAbs belongs to Group A (Table 2) and reacts only with undenatured and intact protein antigens. Structural epitopes formed by tertiary structures are formed by interactions between distal amino acids. Tertiary structures, which are generally formed by interactions between distal amino acids, are more thermodynamically unstable than secondary structures [26], and structural alterations of even a portion of a protein region can affect the overall protein structure. The mAbs belonging to group A were used for immunoprecipitation and reacted only with MIHS and SAST. These mAbs were considered class 1 tertiary structure recognition antibodies because they did not react when partially denatured, as demonstrated by ELISA and WB. Post-translational modifications and amino acid substitutions can alter the overall tertiary structure. Tertiary structure recognition antibodies can be used as sensors to detect these modifications and amino acid substitutions.

### Class 2: mAbs for the epitopes of partially denatured structures

This class includes many mAbs that are generally considered useful and belong to groups B, C, and D as presented in Table 2. This class of mAbs can recognize the functional structure by immunoprecipitation; however, mAbs that recognize partially denatured epitopes of the protein structure have different reactivities depending on the detection method. In ELISA, the protein is fixed to the plate bottom in the hydrophobic region of the protein; therefore, it is considered that some changes have occurred in the protein structure due to fixation to the bottom. In WB, the reactivity between the antigen protein and the antibody was examined, in which the fragile structure was denatured by heat and SDS. As some protein secondary structures are heat-stable and some are unstable [27], it is considered that some of the protein structures used in WB maintain the conformational epitopes; however, a significant portion is denatured. The fact that mAbs in groups B, C, and D showed diverse reaction patterns in ELISA and WB was expected because of the structural changes in different protein regions depending on the method.

### Class 3: mAbs for the epitopes of denatured structures

This class of mAbs does not react with conformational epitopes of the protein but recognizes only denatured protein antigens. As class 2 mAbs that have various applications involving denaturing can also be obtained, there is little need for this class of antibodies. No mAbs of this class could be obtained from the MS clones reported in this study.

By combining MIHS and SAST screening and the short immunization period (3 weeks) reported herein, all processes from the first immunization to generating the desired hybridoma clone could be completed within 35 days. Short-term immunization produces antibodies with low binding affinity owing to insufficient maturation of B cells. However, this method efficiently generated antibodies with high binding affinity and specificity in a relatively short time. Moreover, as expected, we confirmed that immunoprecipitation and dSAST can selectively acquire conformational epitope-specific mAbs. For hybridoma technology, the higher the efficiency of cell fusion between antigen-sensitized B cells and myeloma, the greater the chance of obtaining good antibodies. Cell fusion using the electrofusion method is 10–100 times more efficient (~ 3 × 10^5^ hybridomas can be obtained from 2 × 10^8^ B cells) than that using polyethylene glycol. Under such conditions, it is extremely difficult to select antibody-producing cells from hybridomas by ELISA or single-colony picking; however, MIHS can detect positive signals from a vast number of fused cells using FCM. The number of positive cells selected by MIHS (typically less than 10^3^ cells) allowed SAST to be used for secondary screening. These results indicate that mAbs targeting conformational epitopes of protein structures can be quickly and effectively generated from many hybridomas using the MIHS and SAST approaches. Therefore, the novel mAb screening strategy reported here could accelerate the development of antibody drugs and tests. The limitation of this method is that mAbs cannot be produced using insoluble antigens or multiple transmembrane protein antigens, and future technological improvements are needed to make this possible.

## Conclusions

In this study, a combination of the MIHS and SAST methods was used to screen mAbs that selectively recognize three dimensional structures of protein antigens. Approximately 25% of the mAbs obtained using this method were antibodies that did not react to slight changes in protein structure, suggesting that they recognized stereostructural epitopes comprising distal amino acids. Furthermore, the intensity of fluorescent labeling and the BCR of the hybridoma could be used as indicators to select antibodies with strong binding constants at the time of MIHS screening.

## Supplementary Information

**Additional file 1: Table S1.**

**Additional file 2: Fig. S1.** Confirmation of biotin labeling of the target protein. Samples of non-biotinylated EGFP-BAPHis and biotinylated EGFP-BAPHis with or without streptavidin were prepared as described in the Materials and Methods, and SDS-PAGE was used to show the degree of biotinylation of the antigen protein. Comparing lanes 4 and 5, no bands with the expected molecular weight of EGFP-BAPHis (approximately 28 kDa) were observed in Lane 5, in which streptavidin was added to biotinylated EGFP-BAPHis. Alternatively, two bands (indicated with black arrows) were identified at positions higher than those of streptavidin, confirming that the protein was efficiently labeled by biotin. Lane 1, Streptavidin; Lane 2, Non-biotinylated EGFP; Lane 3, Biotinylated EGFP; Lane 4, Streptavidin + Non-biotinylated EGFP; Lane 5, Streptavidin + Biotinylated EGFP. **Fig. S2.** Confirmation of reproducibility of the SAST method. To confirm the reproducibility of the proposed method, the same procedure described in the Materials and Methods section was performed twice using culture supernatants from 94 anti-EGFP hybridoma clones generated in a previous study (15). The horizontal and vertical axes represent the absorbances of the first and second experiments, respectively. The linear approximation equation is shown in the Fig. **Fig. S3.** FCM profile of the first screening using MIHS from the fusion mixture. The cells cultured after the fusion of B cells from the spleens of mice immunized with EGFP and myeloma cells were examined via FCM. Compared with the FCM profile of the unlabeled fused cells (H-LI), approximately 1% of the cells was in the positive area (more than 3 × H-LI). The positive area is enlarged and colored green. **Fig. S4.** Sensograms of other mAbs not provided in Fig. 6a.

## Data Availability

The dataset supporting the conclusions of this article is included within the additional file.

## Abbreviations

BAP: Biotin acceptor peptide tag
BCR: B-cell receptor
dSAST: Denaturing SAST
EGFP: Enhanced green fluorescent protein
ELISA: Enzyme-linked immunosorbent assay
EW: ELISA-western screening
HRP: Horse radish peroxidase
mAb: Monoclonal antibody
MIHS: Membrane-type immunoglobulin-directed hybridoma screening
MS: MIHS-SAST screening
MW: MIHS-western screening
PBS: Phosphate buffered saline
SAST: Streptavidin-anchored ELISA screening technology
SDS-PAGE: Sodium dodecyl sulfate–polyacrylamide gel electrophoresis
TBST: Tris-buffered saline with tween 20
TMB: 3,3',5,5'-Tetramethylbenzidine
WB: Western blotting

## References

[1] Basu K, Green EM, Cheng Y, Craik CS (2019). Why recombinant antibodies - benefits and applications. Curr Opin Biotechnol.

[2] Hillman Y, Lustiger D, Yariv Y (2019). Antibody-based nanotechnology. Nanotechnology..

[3] Köhler G, Milstein C (1975). Continuous cultures of fused cells secreting antibody of predefined specificity. Nature.

[4] González-Fernández Á, Bermúdez Silva FJ, López-Hoyos M, Cobaleda C, Montoliu L, Del Val M, Leech K (2020). Non-animal-derived monoclonal antibodies are not ready to substitute current hybridoma technology. Nature Meth.

[5] Parray HA, Shukla S, Samal S, Shrivastava T, Ahmed S, Sharma C, Kumar R (2020). Hybridoma technology a versatile method for isolation of monoclonal antibodies, its applicability across species, limitations, advancement and future perspectives. Int Immunopharmacol..

[6] Sorouri M, Fitzsimmons SP, Aydanian AG, Bennett S, Shapiro MA (2014). Diversity of the antibody response to tetanus toxoid: comparison of hybridoma library to phage display library. PLoS One..

[7] Pedrioli A, Oxenius A (2021). Single B cell technologies for monoclonal antibody discovery. Trends Immunol..

[8] Carbonetti S, Oliver BG, Vigdorovich V, Dambrauskas N, Sack B, Bergl E, Kappe S, Sather DN (2017). A method for the isolation and characterization of functional murine monoclonal antibodies by single B cell cloning. J Immunol Methods..

[9] Smith MJ, Packard TA, O’Neill SK, Hinman RM, Rihanek M, Gottlieb PA, Cambier JC (2017). Detection and enrichment of rare antigen-specific B cells for analysis of phenotype and function. J Vis Exp..

[10] Sun Y, Ho M (2020). Emerging antibody-based therapeutics against SARS-CoV-2 during the global pandemic. Antib Ther..

[11] Mitra S, Tomar PC (2021). Hybridoma technology; advancements, clinical significance, and future aspects. J Genet Eng Biotechnol..

[12] Parks DR, Bryan VM, Oi VT, Herzenberg LA (1979). Antigen-specific identification and cloning of hybridomas with a fluorescence-activated cell sorter. Proc Natl Acad Sci U S A..

[13] Price PW, McKinney EC, Wang Y, Sasser LE, Kandasamy MK, Matsuuchi L, Milcarek C, Deal RB, Culver DG, Meagher RB (2009). Engineered cell surface expression of membrane immunoglobulin as a means to identify monoclonal antibody-secreting hybridomas. J. Immunol Methods..

[14] Akagi S, Nakajima C, Tanaka Y, Kurihara Y (2018). Flow cytometry-based method for rapid and high-throughput screening of hybridoma cells secreting monoclonal antibody. J Biosci Bioeng..

[15] Sakaguchi A, Nakajima C, Sawano A, Tanaka Y, Kurihara Y (2021). Rapid and reliable hybridoma screening method that is suitable for production of functional structure-recognizing monoclonal antibody. J Biosi Bioeng..

[16] Liu H, White J, Crawford F, Jin N, Ju X, Liu K, Jiang C, Marrack P, Zhang G, Kappler JW (2015). A rapid method to characterize mouse IgG antibodies and isolate native antigen binding IgG B cell hybridomas. PLoS One..

[17] Sakashita K, Tsumoto K, Tomita M (2022). Advanced hybridoma technology for selective production of high-affinity monoclonal antibodies through B-cell receptors. J Immunol Methods..

[18] Alhajj M, Farhana A (2021). Enzyme linked immunosorbent assay.

[19] Kimura R, Yoda A, Hayashizaki Y, Chiba J (2010). Novel ELISA using intracellularly biotinylated antigen for detection of antibody following DNA immunization. Jpn J Infect Dis.

[20] Verma V, Kaur C, Grover P, Gupta A, K Chaudhary V (2018). Biotin-tagged proteins: Reagents for efficient ELISA-based serodiagnosis and phage display-based affinity selection. PLoS One..

[21] Fairhead M, Howarth M (2015). Site-specific biotinylation of purified proteins using BirA. Methods Mol Biol..

[22] Kuzuya A, Numajiri K, Kimura M, Komiyama M (2008). Single-molecule accommodation of streptavidin in nanometer-scale wells formed in DNA nanostructures. Nucl Acid Symp Ser..

[23] Ainavarapu SRK, Brujic J, Huang HH, Wiita AP, Lu H, Li L, Walther KA, Carrion-Vazquez M, Li H, Fernandez JM (2007). Contour length and refolding rate of a small protein controlled by engineered disulfide bonds. Biophysical J..

[24] Sela M, Schechter I, Borek F (1967). Antibodies to sequential and conformational determinants. Cold Spring Harbor Symp Quant Biol.

[25] Angeletti RH (1999). Design of useful peptide antigens. Peptide antigens. J Biomol Tech.

[26] Cauchy M, D'Aoust S, Dawson B, Rode H, Hefford MA (2002). Thermal stability: a means to assure tertiary structure in therapeutic proteins. Biologicals.

[27] Benjwal S, Jayaraman S, Gursky O (2007). Role of secondary structure in protein-phospholipid surface interactions: reconstitution and denaturation of apolipoprotein C-I:DMPC complexes. Biochem.

