# Supplementary material for: Rapid, simple, and effective strategy to produce monoclonal antibodies targeting protein structures using hybridoma technology
Source: J Biol Eng. 2023 Mar 30;17:24. doi: 10.1186/s13036-023-00345-9 (PMC10061363; doi:10.1186/s13036-023-00345-9)
Supplement: Supplementary file 2 — Additional file 2: Fig. S1. Confirmation of biotin labeling of the target protein. Samples of non-biotinylated EGFP-BAPHis and biotinylated EGFP-BAPHis with or without streptavidin were prepared as described in the Materials and Methods, and SDS-PAGE was used to show the degree of biotinylation of the antigen protein. Comparing lanes 4 and 5, no bands with the expected molecular weight of EGFP-BAPHis (approximately 28 kDa) were observed in Lane 5, in which streptavidin was added to biotinylated EGFP-BAPHis. Alternatively, two bands (indicated with black arrows) were identified at positions higher than those of streptavidin, confirming that the protein was efficiently labeled by biotin. Lane 1, Streptavidin; Lane 2, Non-biotinylated EGFP; Lane 3, Biotinylated EGFP; Lane 4, Streptavidin + Non-biotinylated EGFP; Lane 5, Streptavidin + Biotinylated EGFP. Fig. S2. Confirmation of reproducibility of the SAST method. To confirm the reproducibility of the proposed method, the same procedure described in the Materials and Methods section was performed twice using culture supernatants from 94 anti-EGFP hybridoma clones generated in a previous study (15). The horizontal and vertical axes represent the absorbances of the first and second experiments, respectively. The linear approximation equation is shown in the Fig. Fig. S3. FCM profile of the first screening using MIHS from the fusion mixture. The cells cultured after the fusion of B cells from the spleens of mice immunized with EGFP and myeloma cells were examined via FCM. Compared with the FCM profile of the unlabeled fused cells (H-LI), approximately 1% of the cells was in the positive area (more than 3 × H-LI). The positive area is enlarged and colored green. Fig. S4. Sensograms of other mAbs not provided in Fig. 6a. [file 13036_2023_345_MOESM2_ESM.pptx]

## Slide 1
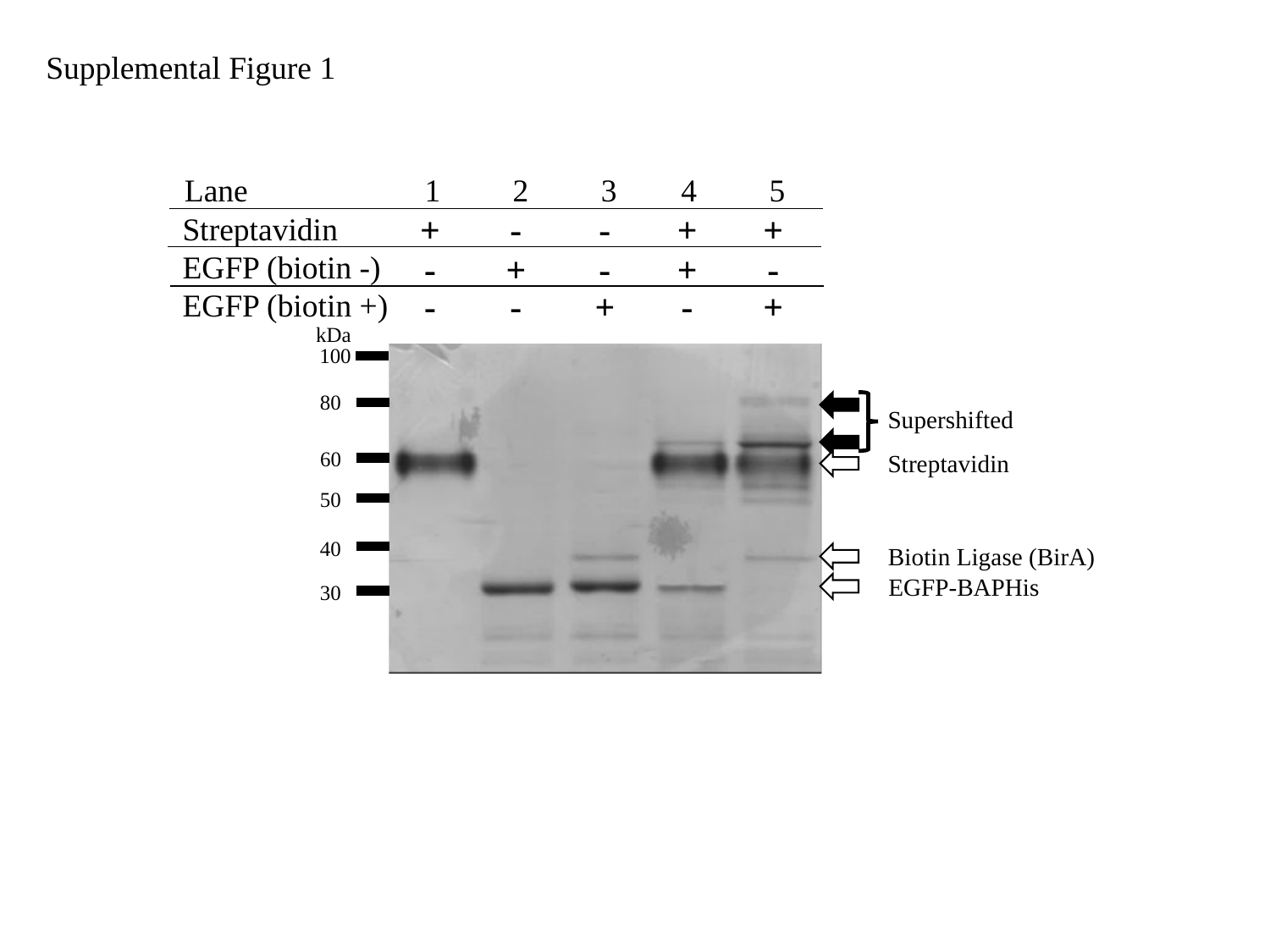

Supplemental Figure 1
Lane 1 2 3 4 5
+
-
-
+
+
Streptavidin
EGFP (biotin -)
EGFP (biotin +)
-
+
-
+
-
-
-
+
-
+
kDa
100
80
Supershifted
60
Streptavidin
50
40
Biotin Ligase (BirA)
EGFP-BAPHis
30

## Slide 2
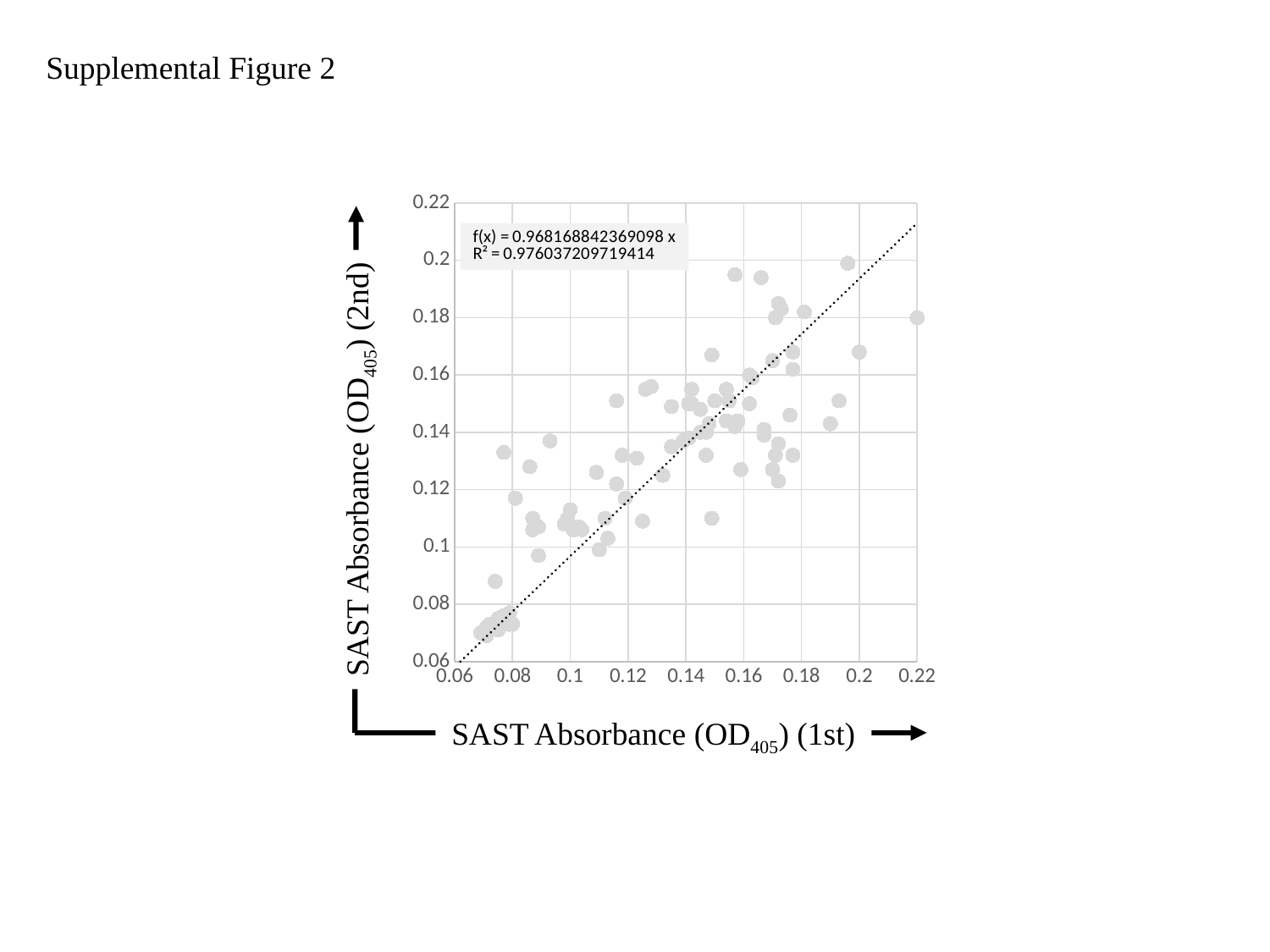

Supplemental Figure 2
### Chart
| Category | |
|---|---|SAST Absorbance (OD405) (2nd)
SAST Absorbance (OD405) (1st)

## Slide 3
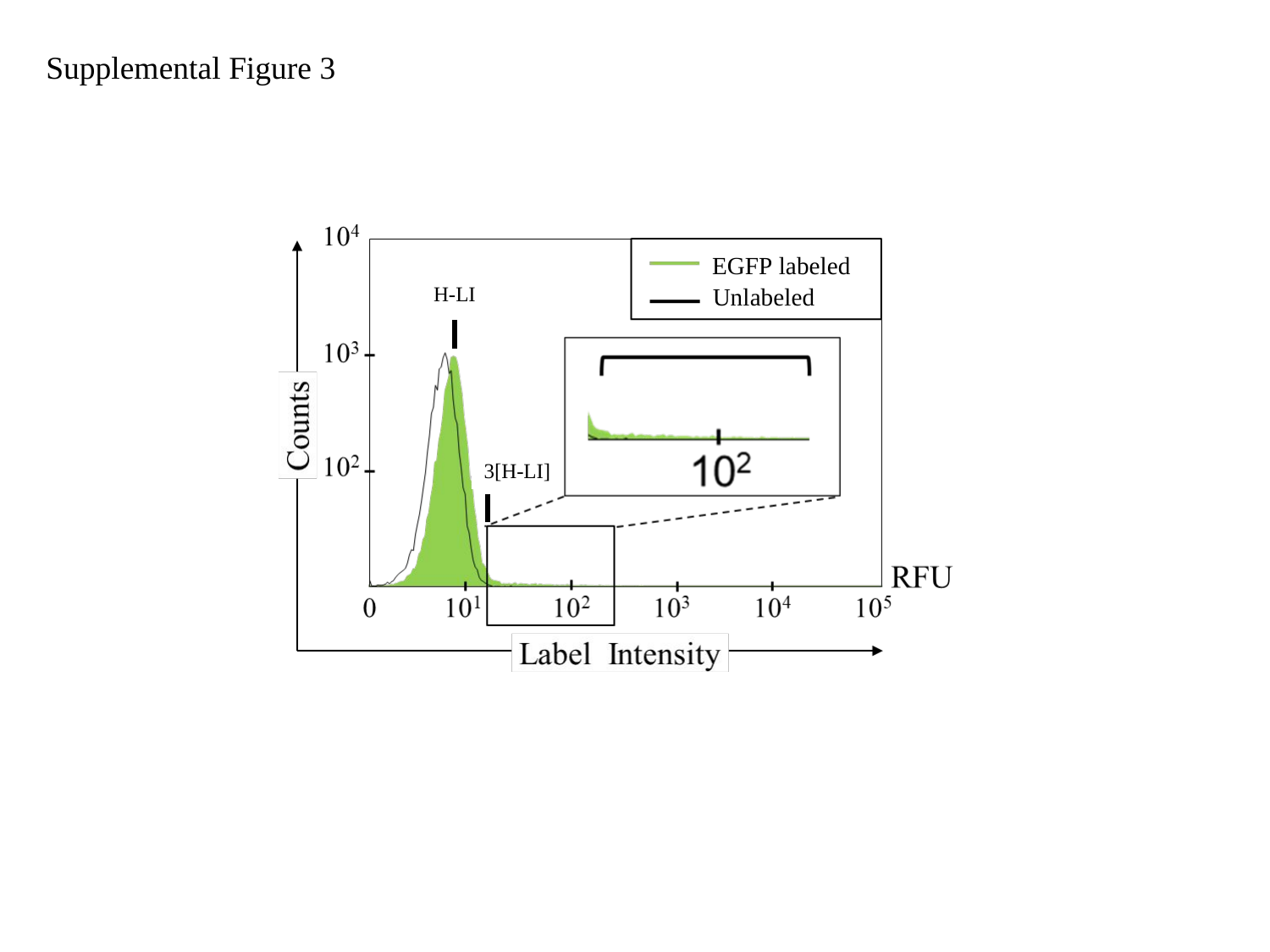

Supplemental Figure 3
EGFP labeled
H-LI
Unlabeled
H-LI
3[H-LI]
3 x [H-LI]

## Slide 4
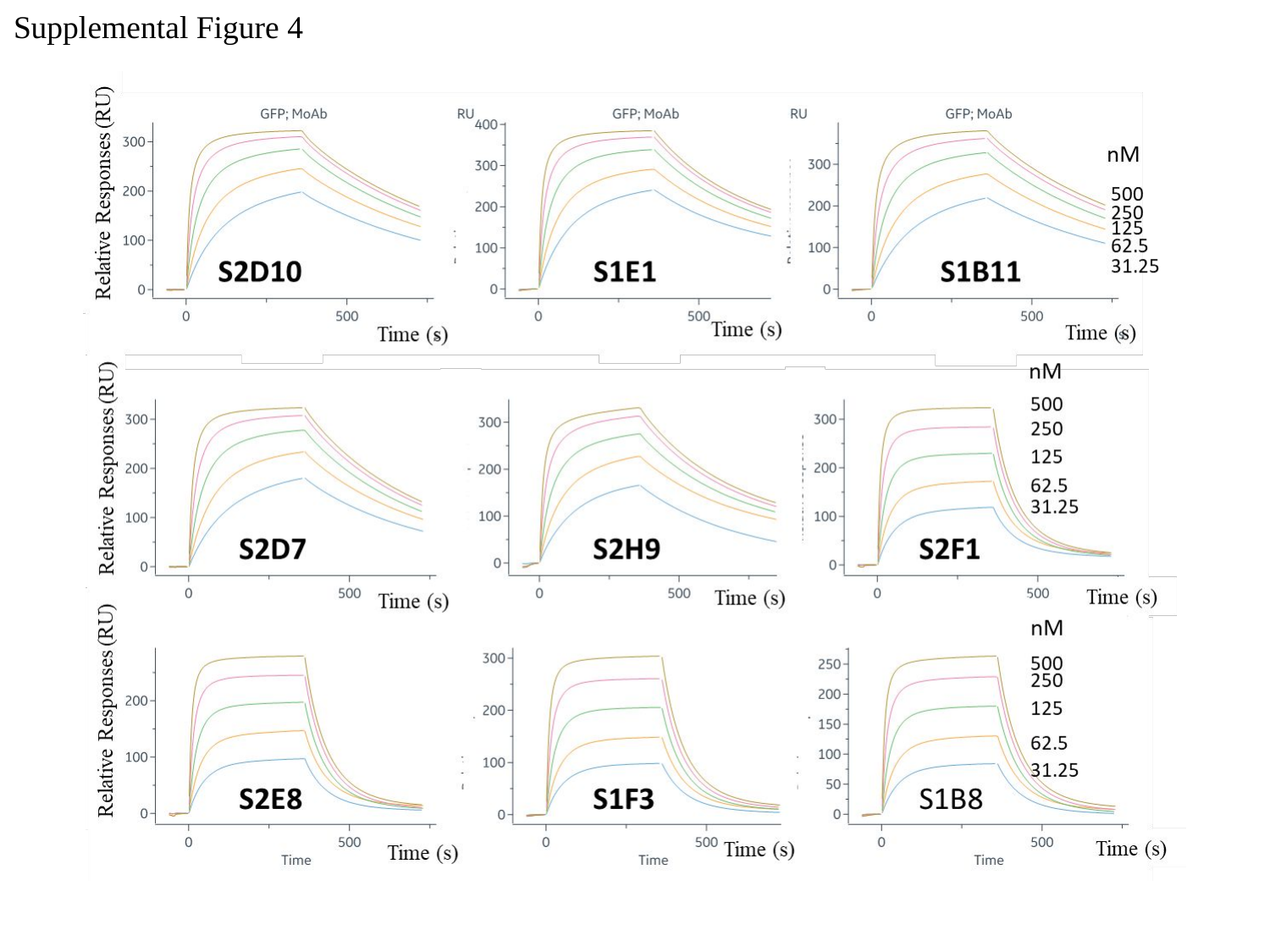

Supplemental Figure 4
